# Implementation of Neonatal Screening Program for Congenital Hypothyroidism in Eastern Morocco

**DOI:** 10.3390/ijns11030055

**Published:** 2025-07-17

**Authors:** Fatima Wahoud, Samia Essadki, Khadija Zirar, Rajae Lamsyah, Wissam Hajjaji, Rim Amrani

**Affiliations:** 1 Maternal Child and Mental Health Research Laboratory, Faculty of Medicine and Pharmacy of Oujda, Mohammed First University, Oujda 60049, Morocco; khadija.zirar@ump.ac.ma (K.Z.); amranirim@yahoo.fr (R.A.); 2 Higher Institute of Nursing Professions and Health Techniques, Oujda 60000, Morocco; s.essadki@ump.ac.ma (S.E.); w.hajjaji@ump.ac.ma (W.H.); 3 Higher Institute of Nursing Professions and Health Techniques, Fez 30050, Morocco; rajae.lamsyah@usmba.ac.ma

**Keywords:** congenital hypothyroidism, newborn screening, eastern Morocco

## Abstract

Congenital hypothyroidism (CH) is one of the major preventable causes of intellectual disability. This study evaluates the incidence of CH through a newborn screening (NBS) program in eastern Morocco. A descriptive cross-sectional design was used and heel prick blood samples were collected on blotting paper to measure Thyroid-Stimulating Hormone (TSH) using an immunofluorimetric assay. 4062 newborns were screened (51.3% male, 48.7% female). TSH levels significantly varied by age: newborns sampled before 24 h had a higher median TSH (3.7 µU/mL [0.10–28.90]) compared to those sampled at 24 h or more (2.1 µU/mL [0.10–32.30]; *p* < 0.001). Using age-specific cut-off values, 18 suspected CH cases were recalled (recall rate: 0.44%). Among the 16 cases who completed confirmatory testing, 4 had transient hyperthyrotropinemia (HTT), characterized by mildly abnormal serum TSH and T4 levels that normalized spontaneously after few months without treatment. Three cases were diagnosed with CH confirmed at birth with markedly elevated serum TSH concentrations and significantly reduced T4 levels. Consequently, the birth prevalence of CH confirmed at birth was 1:1354 live births. The median preanalytical delay was 6 days (IQR: 3–12) and the TSH result turnaround was 8 days (IQR: 5–15), potentially affecting timely intervention. This first report from eastern Morocco confirms the relevance of neonatal screening but highlights delays that must be addressed to enhance early diagnosis and management.

## 1. Introduction

Congenital hypothyroidism (CH) is a thyroid hormone deficiency presenting at birth, and it is the most common preventable cause of intellectual disability [1]. Thyroid hormones regulate the function of most organ systems and play a crucial role in children’s normal growth and neurodevelopment. Indeed, CH can cause cardiological, neurological, gastrointestinal, and metabolic dysfunctions if not diagnosed and treated. CH may be of thyroidal (primary CH) or central origin (secondary and tertiary hypothyroidism). Primary CH can be caused by thyroid dysgenesis or thyroid dyshormonogenesis [2]. Recent studies have shown that, with the increasing incidence of primary CH, the prevalence of thyroid dysgenesis and thyroid function defects has changed with around 60% thyroid dysgenesis and 40% functional defects [3].

Most newborns with CH have no clinical signs, and late diagnosis leads to the most severe outcomes, particularly irreversible intellectual disability. Therefore, newborn Thyroid-Stimulating Hormone (TSH) screening is one of the best, most cost-effective tools for preventing intellectual disability in the population, enabling prompt treatment with levothyroxine [4]. In developed countries, the introduction of NBS has largely eliminated neurodevelopmental impairment from CH. However, implementing such a program remains challenging in other countries [5].

For the first time, we carried out a regional NBS study based on measuring TSH in heel prick blood on blotting papers to assess the birth prevalence of CH in eastern Morocco.

## 2. Materials and Methods

This is a descriptive cross-sectional study designed to assess the prevalence of CH in newborns, the TSH turnaround time (TAT) and screening timeframes.

All newborns delivered in eastern Morocco and for whom written parental consent was obtained were included in this study. Newborns with incomplete data were excluded.

This study was conducted in eight public maternity wards and three neonatal units of eastern Morocco from March 2024 to December 2024. Newborn heel prick blood samples were collected on labeled blotting papers (PerkinElmer 226, Waltham, MA, USA), and blood samples were dried and sent to the collection point of the Medical Analysis Laboratory at Al Farabi Regional Hospital (ARH) of Oujda for analysis to minimize measurement bias. Furthermore, all suspected CH cases were recalled by telephone at ARH for a venous TSH and free T4 (FT4) quantification as a confirmatory test and underwent a clinical examination by qualified health workers (midwives or physicians) to collect anthropometric data (weight), clinical data (CH symptoms and also other pathologies observed in newborns), and demographic data (age at screening and sex) using a questionnaire.

Quantitative determination of the TSH in the dried blood was performed using an immunofluorimetric method DELFIA Neonatal hTSH kit (PerkinElmer, Waltham, MA, USA) according to manufacturer’s instructions. Age-related blood TSH cutoffs were applied to report suspected CH. It is necessary to specify that a cutoff of 20 μU/L blood was used for newborns whose ages were less than or equal to 24 h. Conversely, a cutoff of 15 μU/mL was used for those aged more than 24 h old. These thresholds are based on the national recommendations established by the Moroccan Ministry of Health for neonatal CH screening protocols.

Two distinct situations have been defined based on the results of the confirmatory test. Newborns with mildly elevated or borderline serum TSH and/or T4 levels during the initial confirmatory test, which normalized spontaneously without any medical intervention, were classified as having transient HTT. In contrast, newborns with significantly elevated TSH levels and clearly low T4 concentrations, consistent with primary hypothyroidism, were considered as having CH confirmed at birth.

To assess the timeliness of the CH screening program, we collected dates of birth, sampling, analysis, and result transmission. Dates were collected from medical records to avoid performance bias. The screening program was divided into two phases: Phase 1, from heel prick blood sampling to TSH analysis, and Phase 2, from analysis to the transmission of results. The turnaround time (TAT) was defined as the interval between the sample collection and the transmission of TSH results. For each newborn we calculated TAT, Phase 1, and Phase 2 timeframes.

### Statistical Analysis

All data were imported into SPSS software version 25 (IBM Corp., Armonk, NY, USA) for analysis. Descriptive statistics were used to summarize the characteristics of the newborn population, including general data (age, sex, sampling sites, and screening locations), anthropometric and clinical parameters (gestational age, weight, length, symptoms, and associated conditions), and TSH levels. The Kolmogorov–Smirnov test was applied to assess the normality of distribution for continuous variables. As most quantitative data, including TSH values, were not normally distributed, they were expressed as medians with interquartile ranges [Q1–Q3 or IQR], and comparisons were conducted using non-parametric tests. The Mann–Whitney U test was used to compare continuous variables between two independent groups (e.g., TSH levels in newborns sampled before vs. after 24 h). The Kruskal–Wallis test was used for comparing continuous variables across more than two groups (e.g., TSH levels by gestational age category). The Chi-square test Pearson or Fisher’s exact test was used to compare categorical variables, depending on expected frequency distributions. The results were considered as statistically significant when *p* < 0.05.

## 3. Results

### 3.1. Characteristics of the Newborn Population

During the study period, 4062 newborns were enrolled for CH screening, with an almost equal distribution of males and females (sex ratio male to female of 1.05). Over half (57.9%) of the newborns were less than 24 h old, with a median age [Q1–Q3] at screening of 21 [12–36] hours. In our newborn population, 94.5% were born at full term, 4.4% were born prematurely, and only 0.2% were born after term. The birth weight was 3400 [3000–3800] g without any difference between males and females (Table 1).

Most newborns (88.7%) were managed in the maternity center (including both maternity hospitals and maternity home), and 11.3% were in the neonatal unit. Around two thirds (69%) of deliveries took place in urban areas, whereas 31% occurred in rural areas. The screening for CH was carried out in all provinces of eastern Morocco (Table 1).

### 3.2. TSH Variation Among Newborns

The median TSH value for the 4062 newborns was 3.0 [1.8–4.7] µU/mL. A significant difference was observed between the sexes (*p* = 0.01): females had a median TSH of 2.9 µU/mL [1.7–4.5 µU/mL], while males had a slightly higher median of 3.1 µU/mL [1.9–5.0 µU/mL]. The median TSH level was similar in newborns of low birth weight (<2500 g) (3.1 µU/mL [1.9–4.6]) compared with those weighing ≥ 2500 g (3.0 µU/mL [1.8–4.7]). Regarding the term of pregnancy, the median TSH value was higher in post-term newborns (3.9 µU/mL [2.6–5.3]), followed by full-term newborns (3.0 µU/mL [1.8–4.8]) and premature newborns (2.65 µU/mL [1.5–4.1]). Statistical comparisons were performed for all relevant neonatal variables. No significant associations were found between TSH levels and gestational age (term vs. preterm) or birth weight (<2500 g vs. ≥2500 g), and therefore these results were not emphasized in the main analysis. Interestingly, newborns aged less than 24 h had a median TSH of 3.7 µU/mL [2.3–5.7], whereas those aged 24 h or more showed a lower median of 2.1 µU/mL [1.3–3.4]; this difference was statistically significant (*p* < 0.001) (Table 2).

### 3.3. CH Screening in Eastern Morocco

By applying the provided age-related cut-offs, 18 newborns screened positive and were suspected of having CH (recall rate of 0.44%)—12 male and 6 female. Among them, 13 cases were less than 24 h old and 5 cases were aged 24 h or more. Of the 18 cases recalled at ARH for confirmatory testing, 16 presented for analysis. Among them, 4 newborns had mildly elevated or borderline TSH and/or T4 values at the initial confirmatory test, which normalized during follow-up without the need for treatment. These cases were classified as transient hyperthyrotropinemia (HTT), reflecting a temporary and self-limiting disturbance in thyroid function (median TSH 8.89 µU/mL [6.26–9.13 µU/mL]; median T4 20.73 pmol/L [19.27–22.31 pmol/L]), which normalized upon subsequent testing during follow-up without requiring hormone replacement therapy. These cases were classified as transient HTT. In contrast, confirmatory tests identified 3 cases with a diagnosis of CH confirmed at birth, who had a very high TSH and low T4 levels (median TSH = 180 µU/mL [176–196 µU/mL] and median T4 = 3.8 pmol/l [3.78–4.08 pmol/l]). These results showed that the birth prevalence of CH confirmed at birth was 0.07% (3/4062), or 1 case per 1354 live births. For the remaining 9 newborns, confirmatory test results were entirely within normal limits from the outset, indicating false-positive screening results, representing 0.22% (Table 3).

### 3.4. Profile of CH Newborns Suspected of Having CH

The median age at screening was significantly higher in confirmed CH cases (100 h [40–120]) compared to transient HTT cases (9 h [6–11]) and newborns who had a negative confirmatory test (12 h [7–20]). Overall, 62.5% of recalled newborns were screened before 24 h of life. All transient HTT cases were screened before 24 h of life and initially showed mild hormonal abnormalities that later normalized. In contrast, only one out of three newborns with confirmed CH at birth was screened before 24 h; the remaining two were screened later and had clearly abnormal hormone levels requiring treatment.

Regarding birth weight, the median weight was similar in all groups: 3100 g [2500–3400] for newborns with a diagnosis of CH confirmed at birth, 3150 g [2900–3600] for those with transient HTT, and 3120 g [2700–3800] for those with negative confirmatory test. Low birth weight (<2500 g) was reported in 25% of newborns recalled for confirmatory testing, with comparable proportions between those with a diagnosis of CH confirmed at birth and newborns who had a negative confirmatory test, while no transient HTT case had low birth weight.

A thorough examination showed that some clinical signs were observed exclusively in newborns with a diagnosis of CH confirmed at birth; hypotonia and feeding difficulties were present in 100% of cases and mottled skin in 66.7%. Other signs, including cyanosis, constipation, puffy face, and macroglossia, were each only reported in individual cases. These symptoms were not observed in transient HTT cases or negative groups, except for cyanosis and mottled skin, which were present in few cases with negative confirmatory tests (11.1% and 22.2% respectively).

In addition, 6 out of 16 newborns recalled for confirmatory testing presented with associated pathologies including malformation, cardiopathy, and nephropathy. We also observed that comorbidities were not exclusive to cases with a diagnosis of CH confirmed at birth. In fact, 33.3% of newborns with a negative confirmation test had associated pathologies, compared with 66.6% of those with a confirmed CH diagnosis at birth (Table 4).

### 3.5. Assessment of the Timeliness of the CH Screening Program

For the assessment of the timeliness of the screening program, 1119 random samples were analyzed. The overall median turnaround time was 8 days (IQR: 5–15). By dividing the screening program into two phases, we observed that the timeframe of Phase 1 (from heel prick sampling to analysis) was longer, with a median of 6 days (IQR: 3–12) compared to the timeframe of Phase 2 (from analysis to result transmission) between analysis and transmission (1 day). Interestingly, we noted a distinct variation in the turnaround time and timeframes between Oujda, where the Laboratory of ARH is located, and the other screening locations. Indeed, the lowest TAT median was observed in Oujda (5 days, IQR: 4–8), and the highest TAT medians were observed in Nador (20 days, IQR: 16–28) and Driouch (12 days, IQR: 10–14). Similarly, the timeframe of Phase 1 was shorter in Oujda compared to the other locations. However, the timeframes of Phase 2 were closer from one province to another, with a median of 4 days (IQR: 1–4) observed in Jerada and a median of 1 day in Oujda. Finally, we observed that all screening results, available only one day after the analysis, were transmitted within 1 to 40 days after birth, with a median of 9 days (IQR: 6–17) (Table 5).

## 4. Discussion

Congenital hypothyroidism is a thyroid hormone deficiency presenting at birth, and it is the most common preventable cause of intellectual disability. Screening all newborns has been recognized as the most effective method to prevent severe morbidities associated with CH [5,6]. Since 2006, the Moroccan Ministry of Health has drawn up an action plan and protocols for newborn CH screening, and in 2012 it launched a regional program in Rabat-Salé. Gradually, this program extended to other regions of the country [7]. In eastern Morocco, the NS program was officially launched in December 2022. For organizational reasons, sampling did not begin until March 2024. This study is the first experience of regional NBS for CH in eastern Morocco aiming to estimate the birth prevalence of CH and assess the timeliness of this program.

Three strategies are used to detect newborns with CH: a primary T4 test strategy, a primary TSH test strategy (based on measuring either TSH in heel prick blood spot or cord blood TSH), and a combined strategy. Currently, the TSH strategy is the most frequently used in screening programs worldwide [1,5]. The present screening protocol involves measuring heel prick blood TSH. By contrast, a combined strategy has been used in other CH screening studies in Morocco [8,9]. According to the guidelines of the American Academy of Pediatrics, to avoid false positive results, CH screening is recommended between 24 and 72 h when TSH levels stabilize after a normal surge that occurs within hours after birth. However, samples collected before 48 h are preferable to avoid any delay in screening in case of early discharge. In addition, samples collected before 24 h necessitate the use of age-specific TSH reference ranges [5]. Given that early discharges are frequent, we screened all newborns just before discharge, respecting a screening age of 5 days or less, and applied age-related cutoffs for TSH level in accordance with the national standards: 20 µU/L for newborns whose ages are less than or equal to 24 h and 15 µU/mL for those aged more than 24 h old.

The prevalence of preterm and low birthweight varies across geographic regions. The global prevalence of preterm is 11.1%, ranging from 5% in European countries to 18% in African countries [10]. The global prevalence of low-birthweight newborns estimated in 2015 is 14.6%, with an average of 17.3% in Asia and 7.2% in developed countries [11,12]. In this screening program, 4.4% of newborns enrolled were premature and 6.4% were low birthweight, aligning with the literature. In line with national recommendations, repeat TSH screening is advised for preterm and low-birth-weight newborns to detect possible delayed TSH rise. However, in our study, no second screening was conducted for at-risk newborns with normal initial results, which limits our ability to assess CH incidence in this subgroup. In almost all CH screening programs, including the screening program in the Fez region, a female predominance was reported [9,13]. However, no female predominance was found in our study.

Although standard guidelines recommend repeat TSH testing at 15 and 30 days in preterm newborns, twins, and those born to mothers with thyroid disorders, this protocol was not implemented in our cohort. Only newborns with initial TSH above threshold values were recalled for confirmation. Recall rates in different screening programs vary from 0.01 to 13.3% and are generally lower in primary TSH screening programs than in the T4 strategy [14]. The recall rate in our screening program was 0.44%, which is closer to those reported in the Rabat region (0.6%) and the Fez region (0.5%) [8,9]. This higher rate could be explained by lower cutoffs applied here (20–15 mU/µL) and iodine deficiency or excess; the rate can rise from 0.1% in the non-iodine-deficient population to 20% in the iodine-deficient population [15]. Of note, Morocco is considered a moderate iodine-deficiency area [8,9]. Newborns exposed to excess iodine, such as iodine-containing antiseptics, may also develop hypothyroidism [16,17]. Future improvements to the screening program should include systematic follow-up for high-risk newborns to better detect delayed-onset congenital hypothyroidism.

CH is usually sporadic and occurs in 1 in 2000 to 4000 births. In recent decades, an increase in the incidence of CH has been reported in many studies worldwide [18,19]. This increase is thought to be related to a switch in screening strategies, lower TSH cutoffs, changes in ethnic demographics, and an increased number of preterm and low-birthweight newborns screened [3]. Historically, switching from the primary T4 strategy to the primary TSH strategy has led to an increase in the incidence of primary CH [1]. On the other hand, lowering the TSH cutoffs has led to a change in the etiologies of CH, with more mild and transient cases being detected [19,20]. Significant variation in incidence has been observed among ethnicities, with a higher rate in Hispanic (1:1600), Asian (1:1757 to 1:2380), and non-Hispanic white newborns (1:3333) and a lower rate in Black newborns (1:11,000) [21]. A higher incidence has also been reported in multiple pregnancies (10.1: 10,000) [22] and in same-sex twins (1:593) [23,24].

In Morocco, we have no data on the overall birth prevalence of CH, and few studies have been conducted on the topic. The first neonatal screening campaign for CH was carried out in 1996 in the Rabat region, revealing an incidence of 1:1138 [8]. Another study carried out in the Fez region reported an incidence of 1:1952 [9]. In this study, we report a birth prevalence of 1:1354 live births of CH confirmed at birth, which aligns with Moroccan studies. In addition, according to international guidelines on CH, the four newborns with elevated TSH at screening and mildly abnormal or borderline serum TSH and/or T4 values at the initial confirmatory test, which normalized spontaneously without treatment during follow-up, were classified as having transient HTT. These cases are distinct from both confirmed CH and false-positive results, as they reflect a temporary functional disturbance rather than a false alarm or permanent hypothyroidism. Also, the three newborns with high serum TSH and low serum T4 at confirmation cannot be considered cases with permanent CH but rather cases with a diagnosis of CH confirmed at birth, unless a diagnosis of thyroid dysgenesis is provided in the first days of life by means of thyroid scintigraphy and/or ultrasound. In the absence of an ascertained diagnosis of thyroid dysgenesis (agenesis, ectopy, hypoplasia), a re-evaluation of the diagnosis should be performed at 2–3 years of life after withdrawal of the replacement therapy to verify whether CH confirmed at birth is permanent (re-start therapy) or transient (no therapy). It should be noted that, in a related study exploring maternal and neonatal factors affecting TSH levels in the same region, significantly higher rates of elevated TSH were observed in the province of Figuig (OR = 3.878; *p* = 0.024), possibly linked to high consanguinity rates and other contextual variables [25]. Although this current study did not identify statistically significant geographic variations in confirmed CH cases due to the limited number of diagnoses, these findings support the need for further investigation into province-specific risk factors.

Most newborns with CH have few or no clinical manifestations at birth. Furthermore, symptoms of hypothyroidism are non-specific. As a result, up to 60% of newborns are unaware of their conditions [26]. Studies conducted over the last two decades have also shown that many newborns with CH have concomitant congenital abnormalities such as cardiac and musculoskeletal malformations [27]. CH is also very common in Down’s syndrome newborns (1 to 12%) [5]. In Morocco, Oulmaati et al. reported that most newborns with CH had hypotonia (46%), hypothermia (32%), jaundice (34%), lethargy (22%), feeding difficulties (32%), myxedema (15%), macroglossia (15%), dry skin (12%), and wide fontanelle (10%). Moreover, 78% of these newborns had associated pathologies, especially Down’s syndrome, Turner’s syndrome, cardiopathies, and digestive atresia [28]. In our study, the most common symptoms were hypotonia (42.9%) and feeding difficulties (42.9%), followed by mottled skin (28.6%). The other symptoms represented only 14.3%. In addition, three out of seven cases presented with associated pathologies, including malformation, cardiopathy, and nephropathy. These findings align with previous studies and highlight that NBS is the most effective approach to identifying CH cases.

Neonatal screening programs require the perfect coordination of a multidisciplinary team, robust organization, and consistent funding. In our context, the Medical Analysis Laboratory of ARH is the only laboratory in eastern Morocco to perform TSH quantitation on blotting papers, and for technical and logistical reasons, dried blood samples were sent to the collection point of the laboratory, where TSH quantification was carried out once a week. In addition, all results were available the day after analysis. Therefore, timeframes between sampling and analysis, as well as those between analysis and transmission, are crucial for therapy. Our time monitoring showed that the timeframes of screening phases were longer, especially the pre-analytic delay, resulting in an elevated turnaround time. The variability in TAT observed across provinces highlights significant logistical and structural challenges encountered during the initial phase of the screening program. These include the absence of a dedicated transport system for blood samples, particularly from rural areas; the irregular supply of screening materials; and a shortage of trained healthcare staff at collection sites. Furthermore, the regional reference laboratory operated with very limited human resources, which contributed to processing delays. These constraints, especially prevalent in provinces like Nador and Berkane, may lead to delayed confirmatory testing and late initiation of treatment in newborns with elevated TSH levels, which increases the risk of preventable intellectual disability. This finding is alarming and highlights the need to optimize these processes. Indeed, the routing of blotting papers and results transmission requires strong coordination between midwives, service majors, regional intermediaries, and the laboratory. Strengthening the logistics chain, improving supply continuity, and reinforcing the regional laboratory’s capacity are essential to ensure timely and equitable neonatal screening in eastern Morocco. We also suggest that abnormal results should be communicated directly to a designated physician coordinator in each maternity ward or neonatal unit and transferred into the hospital information system.

We are aware that all screened newborns were enrolled in the main public hospitals in each province. Therefore, this program should be implemented in all medical care centers of eastern Morocco to achieve an effective CH screening program. The screening strategy used here could also be a limitation. Each screening strategy has advantages and disadvantages, leading to a different recall rate and CH birth prevalence. Indeed, in the primary TSH approach, central hypothyroidism, hypothyroxinemia, TBG deficiency, and delayed TSH elevation would be missed. By contrast, the T4 approach is more sensitive in detecting rare cases of hypothalamic–pituitary hypothyroidism. However, the TSH approach has a higher specificity with a smaller recall rate than the T4 program. However, while the combined TSH and T4 strategy is the ideal approach, it is not cost-effective [29]. Reducing the cutoff to 5 mU/L in the UK enabled the identification of cases that would not be detected with the recommended cutoffs [30]. Further studies are thus needed to define optimal cutoffs in our newborn population. Along with this, the presence of only one laboratory in the Oriental region, an area of more than 90,000 km^2^, explains why the preanalytical delays are very large. This can delay the handling of confirmed CH cases at the right time. As such, it is preferable to equip each province with a laboratory even if the expenses of screening would be higher, as the consequences of intellectual disability are far more costly.

Furthermore, human errors are inevitable and can lead to invalid results and an increased recall rate. Ongoing training of midwives and technicians should be considered to reduce non-compliant samples and technical errors. However, given the complexity and uniqueness of each context and the specific needs of each user, the transfer of knowledge must be innovative and relevant in the pedagogical strategies used [31].

## 5. Conclusions

To the best of our knowledge, this study provides the first comprehensive insight into CH screening in eastern Morocco, reporting a birth prevalence of 1:1354 live births for CH confirmed at birth. However, distinguishing between transient and permanent forms of CH remains doubtful due to the absence of follow-up tests that accurately describe long-term results.

Although the program supports the relevance of neonatal CH screening in eastern Morocco, our findings highlight significant operational challenges that compromise its overall effectiveness. Further research, including longitudinal cohort studies, is essential to optimize screening protocols. In addition, improving infrastructure and streamlining logistics will be essential to ensure equitable implementation of the neonatal screening program across all provinces.

## Figures and Tables

**Table 1:** General data of newborns enrolled for CH screening.

	Total (*N* = 4062)	Male (*N* = 2085)	Female (*N* = 1977)	*p*-Value
Age at screening (hours) (*N* = 3982)				
Median [Q1–Q3]	21 [12–36]	22 [12–37]	21 [12–34]	0.011 ^a^
<24, *n* (%)	2305 (57.9%)	1139 (55.7%)	1166 (60.2%)	0.004 ^b^
≥24, *n* (%)	1677 (42.1%)	907 (44.3%)	770 (39.8%)	
Term pregnancy (weeks) (*N* = 4023)				
<37, *n* (%)	178 (4.4%)	83 (4.0%)	95 (4.8%)	NS ^b^
37 to 41, *n* (%)	3836 (94.5%)	1975 (95.7%)	1861 (94.9%)	
≥41, *n* (%)	9 (0.2%)	5 (0.2%)	4 (0.2%)	
Weight (grams) (*N* = 3834)				
Median [Q1–Q3]	3400 [3000–3800]	3400 [3050–3850]	3400 [3000–3800]	NS ^a^
≥2500, *n* (%)	3590 (93.6%)	1855 (94.5%)	1735 (93.8%)	NS ^b^
<2500, *n* (%)	244 (6.4%)	129 (6.5%)	37 (6.2%)	
Sampling sites				
Maternity center, *n* (%)	3603 (88.7%)	1815 (87.1%)	1788 (90.4%)	>0.001 ^b^
Neonatal unit, *n* (%)	459 (11.3%)	270 (12.9%)	189 (9.6%)	
Environment				
Urban, *n* (%)	2784 (69.0%)	1441 (69.3%)	1343 (68.5%)	NS ^b^
Rural, *n* (%)	1255 (31.0%)	637 (30.7%)	618 (31.5%)	
Screening locations				
Oujda, *n* (%)	1755 (43.3%)	922 (44.3%)	833 (42.2%)	NS ^b^
Nador, *n* (%)	574 (14.2%)	288 (13.8%)	286 (14.5%)	
Jerada, *n* (%)	438 (10.8%)	227 (10.9%)	211 (10.7%)	
Berkane, *n* (%)	309 (7.62%)	152 (7.30%)	157 (7.96%)	
Driouch, *n* (%)	307 (7.57%)	146 (7.01%)	157 (7.96%)	
Guercif, *n* (%)	268 (6.61%)	147 (7.06%)	121 (6.14%)	
Taourirt, *n* (%)	209 (5.15%)	105 (5.04%)	104 (5.27%)	
Figuig, *n* (%)	195 (4.81%)	96 (4.61%)	99 (5.02%)	

^a^ Mann–Whitney U test, ^b^ Fisher exact/Pearson test, NS: not significant.

**Table 2:** TSH variation among newborns.

	TSH (µU/mL)		*p*-Value
	Median	[Q1–Q3]	
Total (*N* = 4062)	3.0	[1.8–4.7]	
Gender			
Male	3.1	[1.9–5.0]	0.01 ^a^
Female	2.9	[1.7–4.5]	
Age (hours)			
<24 h	3.7	[2.3–5.7]	<0.001 ^a^
≥24 h	2.1	[1.3–3.4]	
Weight (grams)			
≥2500 g	3.0	[1.8–4.7]	NS ^a^
<2500 g	3.1	[1.9–4.6]	
Term of pregnancy (weeks)			
<37	2.6	[1.5–4.1]	NS ^b^
37 to 41	3.0	[1.8–4.8]	
≥41	3.9	[2.6–5.3]	

^a^ Mann–Whitney U test, ^b^ KRUSKAL Wallis test, TSH: Thyroid-Stimulating Hormone, NS: not significant.

**Table 3:** Outcomes of the CH screening program in eastern Morocco.

	Total (*N* = 4062)		Male (*N* = 2085)		Female (*N* = 1977)		*p*-Value ^a^
	*n*	(%)	*n*	(%)	*n*	(%)	
Recall rate	18	(0.44%)	12	(0.58%)	6	(0.30%)	NS
Newborns < 24 h	13	(0.32%)	9	(0.43%)	4	(0.20%)	NS
Newborns ≥24 h	5	(0.12%)	3	(0.14%)	2	(0.10%)	NS
Negative confirmatory test	9	(0.22%)	6	(0.29%)	3	(0.15%)	NS
Transient HTT	4	(0.10%)	2	(0.10%)	2	(0.10%)	NS
CH confirmed at birth	3	(0.07%)	2	(0.10%)	1	(0.05%)	NS

^a^ Fisher exact/Pearson test, NS: not significant, CH: congenital hypothyroidism, HTT: hyperthyrotropinemia.

**Table 4:** Clinical profile of newborns recalled for confirmatory testing after positive CH screening.

	Screen-Positive Newborns (*N* = 16)	Negative Confirmatory Test (*N* = 9)	Transient Hyperthyrotropinemia (*N* = 4)	Confirmed CH at Birth (*N* = 3)
Age at screening (hours)				
Median [Q1–Q3]	13 [8–24]	12 [7–20]	9 [6–11]	100 [40–120]
<24, *n* (%)	10 (62.5%)	5 (55.6%)	4 (100%)	1 (33.3%)
≥24, *n* (%)	6 (37.5%)	4 (44.4%)	0 (0.0%)	2 (66.7%)
Weight (grams)				
Median [Q1–Q3]	3100 [2800–3600]	3120 [2700–3800]	3150 [2900–3600]	3100 [2500–3400]
≥2500 *n* (%)	12 (75%)	6 (66.7%)	4 (100%)	2 (33.3%)
<2500, *n* (%)	4 (25%)	3 (33.3%)	0 (0%)	1 (66.7%)
Clinical signs				
Cyanosis	2 (12.5%)	1 (11.1%)	0 (0%)	1 (33.3%)
Mottled skin	4 (25%)	2 (22.2%)	0 (0%)	2 (66.7%)
Hypotonia	3 (18.8%)	0 (0%)	0 (0%)	3 (100%)
Constipation	1 (6.3%)	0 (0%)	0 (0%)	1 (33.3%)
Feeding difficulties	3 (18.8%)	0 (0%)	0 (0%)	3 (100%)
Puffy face	1 (6.3%)	0 (0%)	0 (0%)	1 (33.3%)
Macroglossia	1 (6.3%)	0 (0%)	0 (0%)	1 (33.3%)
Comorbidities				
Malformations	2 (12.5%)	1 (11.1%)	1(25%)	0 (0%)
Cardiopathies	2 (12.5%)	1 (11.1%)	0 (0%)	1 (33.3%)
Nephropathies	2 (12.5%)	1 (11.1%)	0 (0%)	1 (33.3%)

NS: not significant, CH: congenital hypothyroidism.

**Table 5:** Assessment of the timeliness of the CH screening program.

	Timeframes (Days)							
	Birth to Transmission		TAT		Sampling to Analysis		Analysis to Transmission	
	Median	(IQR)	Median	(IQR)	Median	(IQR)	Median	(IQR)
Total (*N* = 1119)	9	(6–17)	8	(5–15)	6	(3–12)	1	(1–4)
Screening locations								
Oujda (*n* = 427)	6	(4–9)	5	(4–8)	4	(2–6)	1	(1–1)
Nador *(n* = 191)	21	(18–29)	20	(16–28)	18	(14–24)	2	(1–4)
Berkane (*n* = 138)	15	(9–18)	13	(8–16)	10	(6–14)	2	(1–4)
Jerada (*n* = 125)	10	(7–15)	9	(6–11)	6	(3–9)	4	(1–4)
Guercif (*n* = 71)	9	(6–14)	8	(5–11)	6	(4–8)	1	(1–2)
Taourirt (*n* = 69)	7	(4–10)	6	(3–8)	4	(2–6)	1	(1–1)
Figuig (*n* = 57)	8	(5–13)	7	(4–11)	5	(3–7)	1	(1–2)
Driouch *(n* = 41)	13	(10–15)	12	(10–14)	9	(6–11)	4	(2–4)

CH: congenital hypothyroidism, TAT: turnaround time, IQR: interquartile range.

## Data Availability

The data presented in this study are available on request from the corresponding author due to their ongoing use in a complementary analysis currently in progress.

## Abbreviations

CH	Congenital hypothyroidism
TSH	Thyroid-Stimulating Hormone
ARH	Al Farabi Regional Hospital
TAT	Turnaround time
